# Monitoring Fungal Burden and Viability of *Sporothrix* spp. in Skin Lesions of Cats for Predicting Antifungal Treatment Response

**DOI:** 10.3390/jof4030092

**Published:** 2018-08-07

**Authors:** Luisa Helena Monteiro de Miranda, Jéssica Nunes Silva, Isabella Dib Ferreira Gremião, Rodrigo Caldas Menezes, Rodrigo Almeida-Paes, Érica Guerino dos Reis, Raquel de Vasconcellos Carvalhaes de Oliveira, Danuza Salles do Amaral de Araujo, Laerte Ferreiro, Sandro Antonio Pereira

**Affiliations:** 1 Laboratório de Pesquisa Clínica em Dermatozoonoses em Animais Domésticos, Instituto Nacional de Infectologia Evandro Chagas (INI), Fundação Oswaldo Cruz (Fiocruz), Rio de Janeiro 21040-360, Brazil; isabella.dib@ini.fiocruz.br (I.D.F.G.); rodrigo.menezes@ini.fiocruz.br (R.C.M.); ericaguerino@gmail.com (É.G.d.R.); danuzasalles@gmail.com (D.S.d.A.d.A.); sandro.pereira@ini.fiocruz.br (S.A.P.); 2 Laboratório de Micologia, Faculdade de Veterinária, Universidade Federal do Rio Grande do Sul, Porto Alegre 91540-000, Brazil; jessicanunes7@gmail.com (J.N.S.); laerte.ferreiro@ufrgs.br (L.F.); 3 Laboratório de Micologia, INI, Fiocruz, Rio de Janeiro 21040-360, Brazil; rodrigo.paes@ini.fiocruz.br; 4 Laboratório de Epidemiologia Clínica, INI, Fiocruz, Rio de Janeiro 21040-360, Brazil; raquel.vasconcellos@ini.fiocruz.br

**Keywords:** sporotrichosis, cat, fungal burden, itraconazole, potassium iodide

## Abstract

Skin lesions in feline sporotrichosis usually present a high fungal burden, making cats an important source of infection. This study evaluated the fungal burden and isolation in skin lesions of feline sporotrichosis during treatment with itraconazole (ITZ), combined with or without potassium iodide (KI). Treatment-naïve cats with culture-confirmed sporotrichosis and presenting skin ulcers were treated for up to 40 weeks with oral ITZ alone (*n* = 74) or combined with KI (*n* = 56). These cats were submitted to monthly sampling of the same lesion for mycological culture and cytopathology until healing of lesion or up to twelve weeks. The fungal burden was expressed as the mean yeast cell count in three microscopic fields from imprint smears. The fungal burden before treatment was significantly higher in cats in which the lesion persisted and in cases of treatment failure when using ITZ alone. After twelve weeks, the median fungal burden decreased to zero in both treatment protocols, suggesting a potential decrease in the risk of transmission of *Sporothrix* spp. from cats. These findings encourage the early treatment of feline sporotrichosis as a control measure. Moreover, the fungal burden in feline sporotrichosis lesions can be a prognostic indicator and a parameter for choosing appropriate therapeutic regimen.

## 1. Introduction

Sporotrichosis is a subcutaneous mycosis that affects humans and several other animals, particularly cats [1,2], and is found worldwide. Currently, a few dimorphic fungal species of the genus *Sporothrix* are recognized as clinically relevant, including *S. schenckii sensu stricto*, *S. brasiliensis*, and *S. globosa* [3]. The classical transmission occurs through traumatic inoculation of *Sporothrix* spp. from contaminated environmental sources such as soil and plant material. Zoonotic sporotrichosis is acquired through scratches, bites, or contact with exudates of skin lesions from sick cats [2,4].

The incidence of zoonotic sporotrichosis has been increasing in southeastern Brazil for the last two decades, resulting in the largest epidemic of sporotrichosis ever reported [2]. *Sporothrix brasiliensis* is the most virulent species of the *Sporothrix* genus and the causative agent of the emerging outbreak of zoonotic sporotrichosis in Brazil [2,5]. Most human cases of the disease have been linked to transmission from infected cats, and the identification of *S. brasiliensis* in human and feline sporotrichosis cases strongly supports zoonotic transmission [5]. The reason cats are such a successful source of infection is probably related to the high fungal burden in their skin lesions and to the isolation of the fungus from their claws and oral cavity [6,7,8].

Feline sporotrichosis has a wide range of clinical presentations, from a single skin lesion to disseminated lesions and systemic disease [9]. Cats are highly susceptible to infection with *Sporothrix* spp., and they frequently develop severe forms of the disease as well as skin lesions containing large numbers of yeast cells [7].

Despite the key role of cats in zoonotic sporotrichosis, little is known about the dynamics of the fungal burden in their lesions and about their capability to successfully transmit *Sporothrix* spp. after the onset of antifungal treatment. Previous studies have shown that some therapeutic protocols may reduce the fungal burden in sporotrichosis, even when the infection is caused by *S. brasiliensis* [10,11,12,13]. Recently, Souza et al. [13] reported a significant reduction in the fungal burden within 5 to 11 weeks after start of antifungal treatment in cats with sporotrichosis caused by *S. brasiliensis*. However, these authors had evaluated a small number of cats (*n* = 22) treated with itraconazole (ITZ) at only two time points and had used histopathology of biopsied skin samples, which is a more invasive method and does not confirm the viability of the fungus. Cytopathological examination (CPE) is sensitive and, compared to histopathology, has the advantage of being a simple to perform, noninvasive, rapid, and inexpensive method for the diagnosis of feline sporotrichosis. This method is therefore suitable for routine use by trained veterinary practitioners [14,15,16]. Thus, CPE may be a promising alternative for monitoring the dynamics of fungal burden in ulcerated skin lesions of cats with sporotrichosis during antifungal treatment.

It is important to note that treatment of feline sporotrichosis remains a challenge and reports of therapeutic failure and recrudescence are common, even when established protocols with ITZ—the drug of choice—are used [17,18]. Within this context, the combination of potassium iodide (KI) capsules with ITZ for the treatment of feline sporotrichosis has shown high cure rate and has emerged as an effective treatment option [19,20]. However, the fungal burden before and during the treatment with KI combined and ITZ has not been evaluated.

The high risk of transmission of *Sporothrix* spp. from cats, along with the fact that the long and challenging treatment of these animals may discourage the compliance of owners with the therapeutic protocols and recommended care practices, has compromised the success of measures for the prevention and control of the disease. Therefore, understanding the dynamics of fungal burden and viability of *Sporothrix* in skin lesions of cats during treatment with different protocols may contribute to improved strategies for the diagnosis and treatment of sporotrichosis as well as help identify potential indicators of the risk of zoonotic transmission.

## 2. Materials and Methods

This study comprised cats with skin ulcers in which sporotrichosis was confirmed by the isolation of *Sporothrix* spp. in culture. These cats were seen at the Laboratório de Pesquisa Clínica em Dermatozoonoses em Animais Domésticos (Lapclin-Dermzoo)/INI/Fiocruz, Rio de Janeiro, Brazil, between January 2013 and January 2015. The cats considered eligible had not received previous antifungal therapy and were followed up monthly for the twelve weeks of treatment or until the evaluated lesion had healed. The procedures described herein were approved by the Ethics Committee on Animal Use (CEUA)/Fiocruz (L69/2012), approved in 24 September 2012.

### 2.1. Cats

The cats included in this study were submitted for clinical evaluation of general condition, extracutaneous signs, and the time elapse between the onset of clinical signs and the first appointment, as informed by the cat owner. In order to estimate the level of dissemination of cutaneous lesions, the cats were divided into three groups: L1, L2, and L3, according to the distribution of skin lesions. The L1 group consisted of cats with lesions at a single site; group L2 comprised cats with lesions in two noncontiguous sites; and group L3 comprised cats with lesions in three or more noncontiguous sites [9].

### 2.2. Sampling

An ulcerated skin lesion from each cat was selected to collect samples for CPE and fungal culture before the start of antifungal treatment (T0). In cats with multiple skin ulcers, the lesion with the largest diameter was selected. The procedures for sample collection have been previously described by Silva et al. [16].

#### 2.2.1. Cytopathological Examination

Three serial impressions of skin ulcers were prepared on a dry and clean glass slide and then stained using the Quick Panoptic method (Instant Prov Kit; Newprov, Pinhais, Brazil). The slides were microscopically analyzed at 1000× magnification. The CPE tested positive if at least one yeast cell suggestive of *Sporothrix* spp. was detected [16].

The fungal burden was expressed as the mean count of yeast cells in three microscopic fields (1000×), one per impression.

#### 2.2.2. Fungal Culture

The samples for fungal culture were collected from the same lesion as that selected for CPE using a sterile swab. Samples were seeded onto Sabouraud Dextrose Agar and Mycobiotic Agar (Difco, Becton-Dickinson, Sparks, MD, USA), incubated at 25 °C and monitored for four weeks. Microscopic and macroscopic characteristics of the mycelial cultures were evaluated on Potato Dextrose Agar. Dimorphism was confirmed by conversion to the yeast phase on Brain Heart Infusion Agar at 37 °C [21,22].

### 2.3. Follow-Up Procedures

Cats included in this study received one of the following therapeutic regimens: 100 mg oral ITZ/cat/day or 100 mg oral ITZ/cat/day combined with KI (2.5 to 20 mg/Kg/day). The combination treatment regimen has been previously described by Reis et al. [19].

Follow-up sampling was carried out monthly for 12 weeks at one, two, and three months after start of treatment (follow-ups T1, T2, and T3, respectively). During the follow-up appointments, the same procedures for clinical sampling were employed in the same skin lesion as the one previously selected in the first appointment, as long as it was still ulcerated. Healed lesions were not eligible for resampling. Cats that did not attend all follow-up appointments within the 12 weeks of the study were excluded.

After 12 weeks of follow-up, the cats that had not been discharged during this period continued to be monitored clinically and therapeutically at the Lapclin-Dermzoo/INI/Fiocruz for a maximum period of 40 weeks. The criteria for clinical cure previously described by Reis et al. [19] were used, which consisted of complete healing of all skin/mucosal lesions and remission of all clinical signs. Therapeutic failure was defined if no clinical improvement occurred in two consecutive appointments, in the case of clinical worsening at any time, or if clinical cure was not achieved after 40 weeks of treatment.

### 2.4. Statistics Analysis

Data were stored and analyzed using the Statistical Package for the Social Sciences (SPSS) 20.0. Pearson’s chi-square test of independence (pP) was used to verify significant associations between the categorical variables. Fisher’s exact test was applied to compare variables with only two categories.

The nonparametric tests Kruskal–Wallis (pKW) and Mann–Whitney (pMW) were employed to determine the correlation between categorical and continuous independent variables. The Wilcoxon signed rank test (pW) for paired comparisons was employed to verify significant correlations between categorical variables and the fungal burden during treatment. A *p*-value < 0.05 was considered to indicate a statistically significant association in all analyses.

## 3. Results

One hundred and thirty cats with confirmed sporotrichosis were included in this study: 74 cats were submitted to monotherapy with ITZ and 56 to combined therapy with ITZ and KI. The results for each group are described below.

### 3.1. Itraconazole

Most (67.6%) of the cats treated with ITZ alone (*n* = 74) were male, crossbred (95.9%) and in good general condition (87.8%). The median age was 24 months (6–96 months; *n =* 69) and the median interval between the onset of clinical signs (information provided by the owner) and the first appointment was 8 weeks (2–48 weeks). Respiratory signs (sneezing, rhinorrhea, and/or dyspnea) were observed in 24 cats (32.4%). Nonrespiratory extracutaneous signs were seen in 57 cats (77.0%), especially lymph node enlargement (*n =* 30; 40.5%). Twenty-five cats (33.8%) were classified in group L1, 19 (25.7%) in group L2, and 30 (40.5%) in group L3.

Regarding the CPE before the beginning of treatment, cats of group L3 had a higher fungal burden than those of groups L1 (pMW *=* 0.03) and L2 (pMW *=* 0.03). A progressive reduction in fungal burden during the treatment was observed in the three groups, with a significant difference (pW < 0.001) between the first sampling (T0) and the first follow-up (T1) and between the first and second (T2) follow-ups (pW = 0.003).

After 12 weeks of antifungal treatment, healing of the lesion evaluated occurred in 62 cats (83.8%) and the lesion persisted in 12 cats (16.2%). Most of the lesions (*n =* 42, 56.8%) had healed within one month of treatment with ITZ. The median fungal burden, expressed as the mean count of yeast cells in three independent microscopic fields, before treatment was higher (pMW = 0.013) in cats with the persistent lesion after 12 weeks of treatment (Med *=* 98.6) compared to those in which the lesion had healed (Med *=* 15.0).

After 12 weeks of treatment with ITZ, the median fungal burden was zero in cats in which the lesion had persisted (*n =* 12), regardless of the distribution of the lesions; the maximum yeast count at CPE was 21.

Table 1 shows the results of fungal culture and CPE, and Table 2 shows the assessment of fungal burden before and during treatment in the selected skin lesions that remained ulcerated.

### 3.2. Combination of Itraconazole and Potassium Iodide

In the group of cats treated with the combination of ITZ and KI (*n* = 56), most animals were also male (83.9%), crossbred (89.28%), and in good general condition (89.3%). The median age was 24 months, ranging from 6 to 84 months (*n* = 55). The median time between the onset of signs and the first appointment was eight weeks, ranging from 1 to 96 weeks. Respiratory signs (sneezing, rhinorrhea, and/or dyspnea) were observed in 12 cats (21.43%). Nonrespiratory extracutaneous signs were found in 49 animals (87.5%), and lymph node enlargement was the most common (*n* = 47; 83.9%). Ten cats (17.9%) were in group L1, 19 (33.9%) in group L2, and 27 (48.2%) in group L3.

A reduction in fungal burden during treatment was observed in cats from groups L1, L2, and L3. The decrease was significant (pW = 0.003) between the first appointment before the beginning of antifungal treatment (T0) and the first follow-up (T1).

After 12 weeks of antifungal treatment, all evaluated lesions had already healed and no samples were therefore collected at this time point. The lesions of 44 cats (78.6%) had already healed at the first follow-up appointment (T1) and the lesions persisted in 12 cats (21.8%).

Table 3 shows the results of fungal culture and CPE, and Table 4 shows the evaluation of fungal burden before and during treatment in cutaneous lesions that were still ulcerated.

### 3.3. Treatment Outcome

Among the 74 cats treated with ITZ alone, 71 were followed up until the treatment outcome. Clinical cure was observed in 57 (77.0%) animals and therapeutic failure in 14 (18.9%). Three cats were lost to follow-up. The median fungal burdens at T0 and T1 were higher (pMWT0 = 0.022 and pMWT1 < 0.001) in cats in which therapeutic failure occurred (MedT0 = 100.0; MedT1 = 2.0) compared to those that were clinically cured (MedT0 = 13.6; MedT1 = 0.0).

The percentage of cats in which the evaluated lesion had healed or that had tested negative at both fungal culture and CPE was higher (pP < 0.001) in all follow-up appointments among cats that had clinical cure compared to those in which therapeutic failure occurred (Table 5).

There was no therapeutic failure in the group of cats treated with the combination of drugs. Among the 56 cats receiving this therapeutic regimen, clinical cure was observed in 53 (94.6%), regardless of fungal burden before the beginning of the treatment. Three cats (5.4%) were lost to follow-up.

## 4. Discussion

The lack of information about the role of treatment of cats in public health policies to curb zoonotic sporotrichosis has resulted in worthless control actions and in the continuous increase in zoonotic sporotrichosis cases. Within this context, in order to shed some light on these aspects, the present study performed serial assessment of fungal burden and viability of *Sporothrix* spp. in skin lesions of cats with sporotrichosis during treatment with different protocols.

In feline sporotrichosis, multiple ulcerated skin lesions are the most frequent clinical presentation [18]. The evaluation of ulcerated cutaneous lesions before the beginning of treatment showed that cats with more disseminated lesions (group L3) presented higher fungal burdens, as previously described [7]. This finding suggests an inefficient immunity against *Sporothrix* spp. infection in these animals. In fact, experimental models have shown that the efficiency of the immune response will affect the control of fungal burden [23,24,25,26]. Similarly, in humans and dogs, the occurrence of lesions with high fungal burdens is usually associated with immunosuppressive conditions, such as malnutrition, use of steroid, and the presence of underlying diseases, especially those that induce immunosuppression, such as AIDS [27,28,29,30,31,32]. In cats, Miranda et al. [7,33] and Souza et al. [13] have already observed that different immune response patterns determine the intensity of the fungal burden in skin lesions of feline sporotrichosis and that large numbers of fungal cells generally occur in lesions with a poorly organized inflammation. In addition, a negative correlation has been observed between fungal burden and the number of lymphocytes, neutrophils, and macrophages [13].

In the current study, cats with multiple lesions and higher fungal burdens tended to have persistent lesions and a higher risk of clinical failure when treated with ITZ alone. Additionally, persistent positivity in both CPE and fungal culture until the third month of treatment was related to negative outcomes in cats under ITZ monotherapy. Souza et al. [13] also observed that high fungal burdens in skin lesions before treatment with ITZ were linked to treatment failure as well as a longer time of wound healing in cats. Thus, the follow-up of fungal burden and fungal viability in skin lesions may identify cats that are refractory to ITZ and for which therapeutic alternatives should be considered. Since CPE is a sensitive, easy to perform, non-invasive, rapid, and inexpensive method for the diagnosis of feline sporotrichosis, it is suitable for routine use by trained veterinary practitioners to evaluate antifungal therapy [14,15,16].

The occurrence of positive CPE result and a negative mycological culture after the first month of treatment may be due to the deleterious effect of antifungal treatment on fungal growth in culture or due to incorrect procedures of sample collection or transportation [34,35]. On the other hand, a negative CPE result and a positive mycological culture may be related to the significant reduction in fungal burden during this period. Thus, the application of more than one method may be useful for the diagnosis of sporotrichosis in cats that have been already treated.

The complete healing of the evaluated lesions occurred earlier in cats treated with ITZ and KI compared to ITZ alone, suggesting that the combination of drugs is more efficient in the healing process and, consequently, in reducing the zoonotic potential. Furthermore, no therapeutic failure was observed in cats submitted to the combined therapy in this study, indicating that in contrast to azole monotherapy, the high fungal burden detected before treatment did not affect the healing process in this group. The combination of KI capsules and ITZ has already proven to increase the cure rate of feline sporotrichosis [19,20]. Although the mechanisms whereby KI mediates this process are still unclear, we demonstrate here that KI not only improved the chance of cure but was also related to the prompt control of fungal burden.

It should be noted that a significant decrease in fungal burden and viability occurred in all lesions evaluated herein, regardless of the therapeutic regimen. This decrease was even observed in lesions that persisted after 12 weeks of follow-up and in lesions of cats with treatment failure. In addition, after twelve weeks of treatment, the median fungal burden in lesions decreased to zero. These results strongly suggest that cats under antifungal therapy do not play a key role in the transmission cycle of *Sporothrix* spp., regardless of the initial burden in their lesions. Moreover, the remarkable reduction in fungal burden and viability in the first month of treatment and fungal clearance in ulcerated skin lesions after 12 weeks of treatment point to an early decrease in the risk of zoonotic transmission. However, the long time that had elapsed between the onset of clinical signs and the beginning of the treatment (Median = 8 weeks) in this study may have led to high fungal burdens in skin lesions, possibly impairing treatment outcome and increasing the potential of zoonotic transmission.

## 5. Conclusions

Taken together, the findings indicate the early initiation of antifungal treatment of feline sporotrichosis as a measure to control the transmission of *Sporothrix* spp. to humans, dogs, and other cats. In addition, the fungal burden in lesions from cats with sporotrichosis can be used as a prognostic indicator as well as a parameter to choose the appropriate therapeutic regimen and to evaluate the clinical response.

## Figures and Tables

**Table 1:** Results of fungal culture and qualitative cytopathological examination from ulcerated skin lesions of cats with sporotrichosis during treatment with itraconazole, Lapclin-Dermzoo/INI/Fiocruz (2013–2015).

Mycological Tests	Results	Number of Cats with Ulcerated Skin Lesions			
		T0 (*n =* 74)	T1 (*n =* 32) *	T2 (*n =* 13) *	T3 (*n =* 12) *
Cytopathological examination	+	65	20	4	3
	−	9	12	9	9
Fungal culture	+	74	17	6	7
	−	0	15	7	5

*n* = total number of cats sampled; + = positive, − = negative; T0 = first appointment, before the beginning of antifungal treatment; T1, T2, and T3 = follow-ups after one, two, and three months of treatment, respectively. * Samples were not collected in 42 cats at T1, 61 cats at T2, and 62 cats at T3 due to the healing of the selected skin lesion.

**Table 2:** Assessment of fungal burden at the cytopathological examination according to the distribution of lesions in cats with sporotrichosis during treatment with itraconazole, Lapclin-Dermzoo/INI/Fiocruz (2013–2015).

Groups		Yeast Cell Count at Cytopathological Examination			
		T0	T1	T2	T3
L1	*n*	25	9	4	4
	Median	9	2	0	0
	Min–Max	0–298	0–50	0–1	0–21
L2	*n*	19	8	1	1
	Median	8.3	0.5	0	0
	Min-Max	0–323	0–17	0	0
L3	*n*	30	15	8	7
	Median	76	1	0	0
	Min–Max	2–413	0–15	0–5	0–11

*n* = number of yeasts; L1, lesions at one site; L2, lesions at two noncontiguous sites; L3, lesions at three or more noncontiguous sites; T0 = first appointment, before the beginning of antifungal treatment; T1, T2, and T3 = follow-ups after one, two, and three months of treatment, respectively; Min = Minimum; Max = Maximum.

**Table 3:** Results of fungal culture and qualitative cytopathological examination from ulcerated skin lesions of cats with sporotrichosis during treatment with itraconazole and potassium iodide, Lapclin-Dermzoo/INI/Fiocruz (2013–2015).

Mycological Tests	Results	Number of Cats with Ulcerated Skin Lesions			
		T0 (*n =* 56) *	T1 (*n =* 12) *	T2 (*n =* 3) *	T3 (*n =* 0) *
Cytopathological examination	+	56	9	2	NP
	−	0	3	1	NP
Fungal culture	+	56	3	0	NP
	−	0	9	3	NP

*n* = total number of cats sampled; + = positive, − = negative; T0 = first appointment, before the beginning of antifungal treatment; T1, T2, and T3 = follow-ups after one, two and three months of treatment, respectively. * Samples were not collected in 44 cats at T1, 53 cats at T2, and 56 cats at T3 due to the healing of selected skin lesion; NP = not performed due to the healing of selected skin lesion.

**Table 4:** Assessment of fungal burden at the cytopathological examination according to the distribution of lesions in cats with sporotrichosis over the treatment with itraconazole and potassium iodide, Lapclin-Dermzoo/INI/Fiocruz (2013–2015).

Groups		Yeast Cell Count at Cytopathological Examination			
		T0	T1	T2	T3
L1	*n*	10	1	0	0
	Median	11.5	0	NC	NC
	Min–Max	1–107	NC	NC	NC
L2	*n*	19	3	1	0
	Median	17.67	2.33	1	NC
	Min–Max	2–177	0–3	1	NC
L3	*n*	27	8	2	0
	Median	13	1.83	0	NC
	Min–Max	2–323	0–3	NC	NC

*n* = number of yeasts; L1, lesions at one site; L2, lesions at two noncontiguous sites; L3, lesions at three or more noncontiguous sites; T0 = first appointment, before the onset of antifungal treatment; T1, T2, and T3 = follow-ups after one, two, and three months of treatment, respectively; Min = Minimum; Max = Maximum. NC = non-calculated due to the healing of selected skin lesion.

**Table 5:** Results of fungal culture and qualitative cytopathological examination for the detection of *Sporothrix* spp. in skin lesions of cats over the first three follow-up appointments, during therapy with itraconazole according to treatment outcome (cure or failure), Lapclin-Dermzoo/INI/Fiocruz (2013–2015).

Follow-Up	Mycological Tests	Results	Treatment Outcome (*N =* 71)	
			Cure	Failure
T1	Fungal culture (*n =* 30)	+	7	10
		−	12	1
	Cytopathological examination (*n =* 30)	+	8	10
		−	11	1
	NP (*n =* 41)	N/A	38	3
T2	Fungal culture (*n =* 11)	+	1	5
		−	2	3
	Cytopathological examination (*n =* 11)	+	0	4
		−	3	4
	NP (*n =* 60)	N/A	54	6
T3	Fungal culture (*n =* 11)	+	0	7
		−	1	3
	Cytopathological examination (*n =* 11)	+	0	3
		−	1	7
	NP (*n* = 60)	N/A	56	4

*N =* Total numbers of cats; *n* = number of cats; + = positive, − = negative; T1, T2, and T3 = follow-ups after one, two, and three months of treatment, respectively. NP = not performed due to the healing of the selected skin lesion; N/A = not applicable because there was no sample collection.
